# Chronic Monobutyl Phthalate Exposure Promotes Anaplastic Thyroid Cancer Progression Through Inflammatory Signaling Dysregulation: Integrated Transcriptomic and Network Toxicology Analyses

**DOI:** 10.3390/biomedicines14081755

**Published:** 2026-08-04

**Authors:** Yu Deng, Songwei Tan, Jinlan Wei, Xingyue Guo, Longqing Hu, Xincai Qu, Jing Zhou

**Affiliations:** 1 Department of Breast and Thyroid Surgery, Union Hospital, Tongji Medical College, Huazhong University of Science and Technology, Wuhan 430030, China; d202482286@hust.edu.cn (Y.D.);; 2 Tongji School of Pharmacy, Tongji Medical College, Huazhong University of Science and Technology, Wuhan 430030, China; tansw@hust.edu.cn; 3 School of Public Health, Tongji Medical College, Huazhong University of Science and Technology, #13 Hangkong Road, Wuhan 430030, China

**Keywords:** monobutyl phthalate (MBP), anaplastic thyroid carcinoma (ATC), network toxicology, transcriptomics, chronic exposure, inflammatory network remodeling

## Abstract

**Background/Objectives:** Chronic exposure to endocrine-disrupting chemicals has been increasingly recognized as a potential contributor to cancer progression. Monobutyl phthalate (MBP), a major metabolite of dibutyl phthalate, is widely detected in human biological samples, yet its long-term impact on anaplastic thyroid cancer (ATC) has not been systematically investigated. **Methods:** CAL-62 cells were continuously exposed to an environmentally relevant concentration of MBP (10 nM) over 3 months to establish a chronic exposure model that mimics long-term environmental exposure. Transcriptomic profiling was integrated with network toxicology to identify key molecular pathways and hub genes, followed by molecular docking and Western blot validation. **Results:** Chronic MBP exposure significantly enhanced cell viability, proliferation, colony formation, and tumorsphere formation, indicating promotion of malignant phenotypes. Transcriptomic profiling revealed extensive molecular remodeling characterized by activation of inflammation-associated pathways, including cytokine–cytokine receptor interaction, IL-17, TNF, and JAK–STAT signaling, accompanied by suppression of p53- and mTOR-related pathways. Integrated analysis identified 57 overlapping KEGG pathways, with IL6 and CSF2 emerging as central hub genes. Molecular docking demonstrated favorable binding affinities between MBP and representative target proteins, including IL6, TP53, CASP3, BCL2, and PPARG. Western blot analysis further confirmed increased IL6 and BCL2 expression together with decreased TP53, CASP3, and PPARG expression following chronic MBP exposure. **Conclusions:** Chronic environmentally relevant MBP exposure promotes ATC malignant progression through coordinated inflammation-associated molecular network remodeling accompanied by suppression of apoptosis-related signaling. Integrating network toxicology with transcriptomic profiling provides an effective systems-level strategy for identifying biologically relevant molecular networks underlying chronic environmental toxicant exposure.

## 1. Introduction

Environmental exposure to endocrine-disrupting chemicals (EDCs) has become an important public health concern and may contribute to the increasing global burden of chronic diseases, including cancer [1]. EDCs are ubiquitously distributed in the environment [2], and humans are continuously exposed through dietary intake, drinking water, inhalation, and dermal contact [3]. Unlike acute toxicological exposure, human exposure to EDCs predominantly occurs at environmentally relevant concentrations over prolonged periods, resulting in persistent biological perturbations [1,4]. Increasing evidence indicates that EDCs not only disrupt endocrine homeostasis but also influence inflammation [5], oxidative stress [6], metabolism [7], and apoptosis [8] through coordinated regulation of multiple molecular targets and signaling pathways. These characteristics highlight the importance of investigating the biological consequences of chronic environmentally relevant exposure, which more closely reflects real-world human exposure scenarios than conventional acute exposure models.

Anaplastic thyroid cancer (ATC) is the most aggressive subtype of thyroid carcinoma, accounting for less than 2% of thyroid cancers but responsible for a disproportionate number of thyroid cancer-related deaths [9,10]. Due to its rapid proliferation, extensive local invasion, early distant metastasis, and resistance to conventional therapies, the prognosis of ATC remains extremely poor, with a median survival of less than one year [11]. These aggressive biological characteristics and limited therapeutic options highlight the importance of investigating additional factors that may contribute to ATC progression beyond established genetic alterations. Although recurrent genetic alterations involving BRAF, RAS, TERT, and TP53 have been extensively characterized, accumulating evidence suggests that environmental factors may also contribute to ATC progression by influencing tumor-associated biological process [12].

Monobutyl phthalate (MBP), the primary bioactive metabolite of dibutyl phthalate (DBP), is one of the most frequently detected phthalate metabolites in human biological samples and is widely used as a biomarker of phthalate exposure [13,14,15,16]. Previous studies have associated MBP exposure with reproductive toxicity, metabolic disorders, immune dysregulation, and thyroid hormone disruption [17]. Experimental evidence has further suggested that MBP may affect apoptosis and inflammatory signaling in different cellular systems [18]. Nevertheless, most available mechanistic studies have relied on acute or relatively high-dose exposure conditions that do not adequately represent chronic environmental exposure [19]. Furthermore, previous investigations have primarily examined individual genes or isolated signaling pathways—such as the AKT/NF-κB/NLRP3 inflammatory axis [20] or the TNF/IL6/STAT3 pathway [21]—providing limited insight into the overall molecular network characteristics associated with chronic MBP exposure. Importantly, epidemiological findings regarding MBP and thyroid cancer risk remain conflicting: while a case–control study in Wuhan reported an inverse association between urinary MBP and thyroid cancer risk [22], meta-analyses have indicated that phthalates (PAEs) as a class are positively associated with increased thyroid cancer risk [23], and the broader literature acknowledges conflicting reports on the association between environmental endocrine disruptors and thyroid cancer [23,24], emphasizing the need for mechanistic investigations under biologically relevant exposure conditions.

Recent advances in transcriptomic profiling enable comprehensive characterization of genome-wide molecular responses induced by environmental toxicants, whereas network toxicology provides a systems-level strategy for predicting toxicological targets and associated biological pathways [12]. These two approaches provide complementary information: transcriptomics reflects the actual biological responses under defined exposure conditions, whereas network toxicology prioritizes potential regulatory networks based on existing biological knowledge. Integrating these approaches therefore offers a more comprehensive framework for identifying biologically relevant molecular networks associated with chronic environmental exposure while improving the biological interpretation of high-throughput transcriptomic data. Notably, a recent study integrating network toxicology and transcriptomic profiling demonstrated that DBP/MBP exposure contributed to metabolic dysfunction-associated steatotic liver disease (MASLD/MASH) via modulation of key targets including ALB, PPARG, HSP90AA1, and PTGS2 [25], highlighting the utility of systems-level approaches in deciphering phthalate-induced toxicity.

In this study, we set up a chronic exposure model using CAL-62, a well-characterized ATC cell line harboring both BRAF V600E and TP53 mutations, which collectively represent the most common genetic backdrop in aggressive ATC and provide a stringent biological context for testing the pro-malignant effects of chronic MBP exposure. We cultured CAL-62 cells with low-dose MBP for three months. Cellular malignant phenotypes were evaluated using cell viability, proliferation, colony formation, and tumorsphere formation assays. Transcriptomic profiling was integrated with network toxicology to systematically characterize molecular alterations associated with chronic MBP exposure, and representative targets were further evaluated by molecular docking and Western blot analysis. Our results demonstrate that chronic environmentally relevant MBP exposure promotes malignant phenotypes in CAL-62 cells and is associated with inflammation-dominant molecular network remodeling accompanied by suppression of apoptosis-related signaling. These findings provide new toxicogenomic evidence for understanding the potential contribution of long-term phthalate exposure to thyroid cancer progression and illustrate the value of integrating computational prediction with experimental validation for investigating the health effects of chronic environmental contaminants.

## 2. Materials and Methods

### 2.1. Chemicals and Reagents

MBP was purchased from Shanghai Macklin Biochemical Co., Ltd. (Shanghai, China) (M814383). Dulbecco’s Modified Eagle Medium (DMEM) was obtained from Gibco (Grand Island, NY, USA) (11330-032). Fetal bovine serum (FBS) was purchased from Lanjieke Technology Co., Ltd. (Shanghai, China) (BL201A). Phosphate-buffered saline (PBS) was obtained from Procell Life Science & Technology Co., Ltd. (Wuhan, China) (PB180327). Trypsin–EDTA and cell freezing medium were purchased from Beyotime Biotechnology (Shanghai, China) (C0222 and C0210, respectively). Dimethyl sulfoxide (DMSO) was obtained from Solarbio (Beijing, China) (D8372). Penicillin–streptomycin was purchased from Merck (Darmstadt, Germany) (HY-K1006). Cell Counting Kit-8 (CCK-8) was obtained from Vazyme (Nanjing, China) (A311-01).

### 2.2. Cell Culture and Establishment of Chronic MBP Exposure Model

CAL-62 human anaplastic thyroid carcinoma (ATC) cells (Cellosaurus accession: CVCL_1112) were selected because they are a well-established in vitro model that retains the aggressive biological characteristics of ATC and has been widely used for mechanistic studies of ATC progression. Cells were cultured in high-glucose DMEM supplemented with 10% FBS and 1% penicillin–streptomycin under standard conditions (37 °C, 5% CO_2_).

MBP was dissolved in sterile ultrapure water to prepare a 10 mM stock solution, filtered, aliquoted, and stored at −20 °C. For experiments, MBP was diluted to a final concentration of 10 nM. Control cells received vehicle only.

For chronic exposure, CAL-62 cells were continuously cultured in medium containing 10 nM MBP for approximately 3 months, during which cells underwent approximately 30 passages. Control cells were maintained under identical culture conditions and passaged for the same duration and number of passages without MBP treatment.

The exposure concentration was selected to mimic environmentally relevant low-dose exposure based on preliminary experiments and previous reports on chronic phthalate exposure.

### 2.3. Functional Assays (Viability, Proliferation, Colony Formation, Tumorsphere Formation)

CCK-8 assay was performed according to the manufacturer’s protocol. Absorbance was measured using a microplate reader and normalized to control. Cell proliferation was assessed using time-course growth curves at days 0, 3, 6, and 9. For colony formation, cells were seeded at low density and cultured for 10–14 days. Colonies (>50 cells) were fixed, stained with crystal violet, and quantified. For tumorsphere formation, cells were cultured in ultra-low attachment plates in serum-free medium. Spheres were imaged and quantified after 7–14 days.

All experiments were performed in triplicate.

### 2.4. RNA Sequencing and Bioinformatic Analysis

RNA was extracted using standard protocols. Libraries were constructed and sequenced on an Illumina platform (Illumina Inc., San Diego, CA, USA).

Raw reads were quality filtered, aligned to the human genome (HISAT2), and quantified as FPKM/TPM.

DEGs were identified using DESeq2 (v1.48.1) an open-source R package maintained by the Bioconductor core team, with |log2FC| > 1 and adjusted *p* < 0.05.

GO and KEGG enrichment analyses were performed using R (v4.3.1). which is developed and maintained by the R Core Team and distributed via CRAN (The Comprehensive R Archive Network). BP, CC, and MF categories were analyzed. Significance was set at adjusted *p* < 0.05.

### 2.5. Network Toxicology Analysis

According to published literature [26], MBP-associated target genes were retrieved from the Comparative Toxicogenomics Database (CTD), TargetNet, and SwissTargetPrediction, whereas ATC-associated genes were obtained from GeneCards and the Online Mendelian Inheritance in Man (OMIM) database. Intersection genes were identified using Venn diagram analysis.

Protein–protein interaction (PPI) networks were constructed using STRING (confidence > 0.4) and visualized in Cytoscape (v3.9.2, an open-source software developed by the Cytoscape Consortium). Hub genes were identified using CytoHubba based on degree centrality.

GO and KEGG enrichment analyses were performed using the Database for Annotation, Visualization and Integrated Discovery (DAVID), which was selected because it is a well-established and extensively validated platform for functional annotation and pathway enrichment analysis and is particularly suitable for relatively small gene sets. A *p* < 0.05 was considered statistically significant.

### 2.6. Integration of Transcriptomic and Network Toxicology Analyses

KEGG pathways were independently enriched from network toxicology targets (130 pathways) and RNA-seq DEGs (89 pathways). Overlapping pathways were identified using Venn analysis, yielding 57 common pathways.

A pathway–target network was constructed in Cytoscape. Degree centrality analysis identified IL6 and CSF2 as key hub genes.

### 2.7. Molecular Docking Analysis

The 3D structure of MBP was obtained from PubChem and energy minimized. Protein structures of TP53, CASP3, BCL2, IL6, and PPARG were retrieved from the Protein Data Bank (PDB). Proteins were prepared using PyMOL (v2.6.0a0; open-source molecular visualization system originally created by Warren L. DeLano, currently maintained and commercialized by Schrödinger, LLC, headquartered at 101 SW Main Street, Suite 1300, Portland, OR 97204, USA) by removing water molecules and ligands. Docking was performed using AutoDock Vina (v1.5.7; developed by the Scripps Research Institute, La Jolla, CA, USA). Grid boxes were individually defined for each protein. The lowest binding energy conformations were selected. Binding affinities were expressed in kcal/mol. Results were visualized using PyMOL and Discovery Studio Visualizer (v 25.1.0.242; developed by Biovia, now part of Dassault Systèmes, headquartered at 175 Wyman Street, Waltham, MA 02451, USA).

### 2.8. Western Blot Analysis

Cells were lysed using RIPA buffer containing protease and phosphatase inhibitors. Protein concentration was measured using a BCA assay. Equal amounts of protein (20 μg) were separated by SDS-PAGE and transferred to PVDF membranes. Membranes were blocked with 5% non-fat milk and incubated overnight at 4 °C with primary antibodies against TP53, CASP3, BCL2, IL6, PPARG, and β-actin. After incubation with HRP-conjugated secondary antibodies, signals were detected using ECL and quantified using ImageJ (v1.54p; an open-source image processing software developed by the National Institutes of Health, Bethesda, MD, USA).

### 2.9. Statistical Analysis

Data are presented as mean ± standard deviation (SD). Statistical analyses were performed using GraphPad Prism (version 10.1.2; GraphPad Software, LLC, located at 7825 Fay Avenue, Suite 230, La Jolla, CA 92037, USA). Student’s *t*-test was used for two-group comparisons. One-way ANOVA was used for multiple comparisons when applicable. *p* < 0.05 was considered statistically significant.

## 3. Results

### 3.1. Chronic Environmentally Relevant MBP Exposure Promotes the Malignant Phenotype of CAL-62 Cells

**Figure 1:**
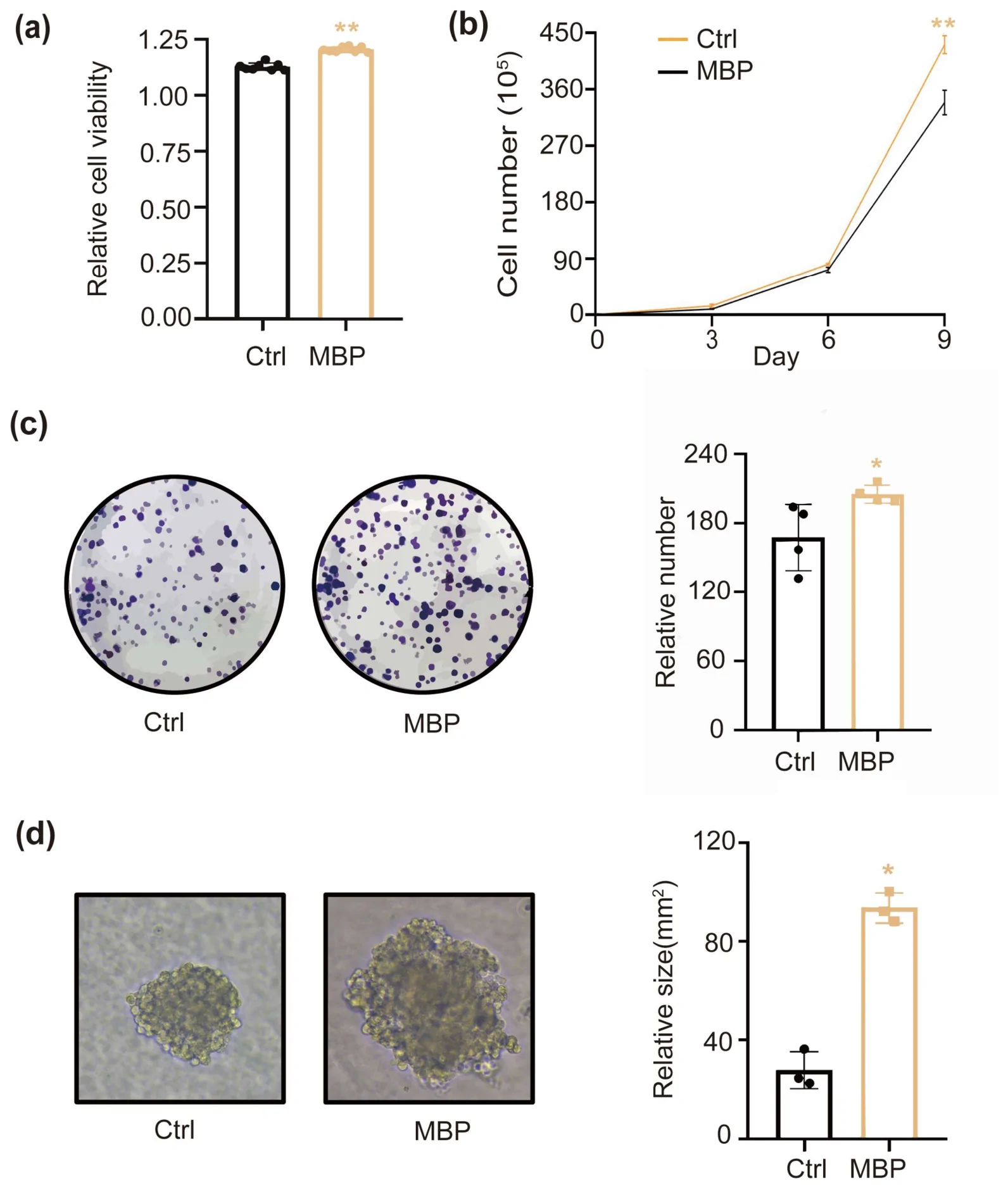
Effects of chronic MBP exposure on the malignant phenotypes of CAL-62 cells. (**a**) Cell viability at 3 months determined by CCK-8 assay. (**b**) Cell proliferation curves monitored from day 1 to day 9. (**c**) Colony formation ability of CAL-62 following chronic MBP exposure. (**d**) Sphere formation capacity of MBP treated cells. Data are presented as mean ± SD. * *p* < 0.05 and ** *p* < 0.01 compared with the control group.

Cell viability analysis showed that chronically MBP-exposed cells exhibited significantly higher viability than control cells (*p* < 0.05; Figure 1a). Consistently, growth curve analysis demonstrated that chronic MBP exposure markedly accelerated cell proliferation, with significantly increased cell numbers observed on days 3, 6, and 9, and the greatest difference detected on day 9 (*p* < 0.05; Figure 1b). To further evaluate the influence of chronic MBP exposure on the malignant characteristics of CAL-62, colony formation and tumorsphere formation assays were performed. Compared with the control group, MBP-treated cells generated significantly more colonies, indicating enhanced clonogenic capacity (*p* < 0.05; Figure 1c). Likewise, chronic MBP exposure markedly increased both the number and size of tumorspheres, suggesting enhanced anchorage-independent growth capacity (*p* < 0.05; Figure 1d). Collectively, these results demonstrated that chronic exposure to environmentally relevant concentrations of MBP significantly enhanced the malignant phenotype of CAL-62, as evidenced by increased cell viability, proliferative capacity, clonogenic potential, and tumorsphere formation ability.

### 3.2. Chronic MBP Exposure Induces Transcriptomic Remodeling Characterized by Inflammatory Signaling Activation

**Figure 2:**
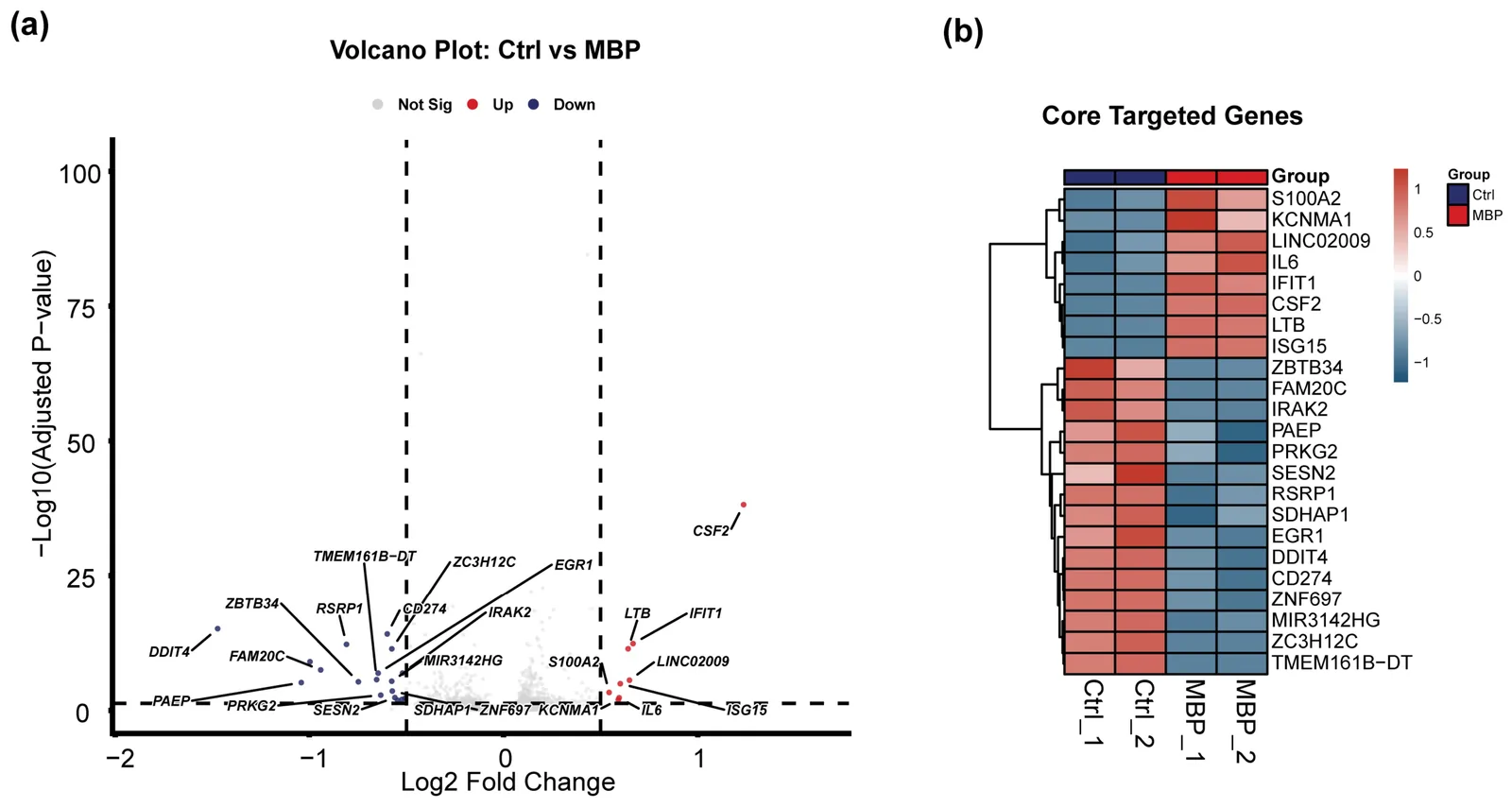
Differential mRNA expression profiles in MBP-treated CAL-62 cells. (**a**) Volcano plot showing the distribution of differentially expressed genes between the control and MBP-treated groups. (**b**) Heat map displaying representative differential genes in the two groups. Upregulated and downregulated genes are shown in red and blue, respectively.

To characterize the molecular alterations associated with the enhanced malignant phenotype induced by chronic MBP exposure, transcriptome sequencing was performed in control and chronically MBP-exposed CAL-62. Differential expression analysis identified 26 significantly differentially expressed genes (DEGs) between the two groups (adjusted *p* < 0.05 and |log_2_ fold change| > 0.5), including 8 upregulated and 18 downregulated genes, as illustrated in the volcano plot (Figure 2a). Representative upregulated genes included IL6, CSF2, ISG15, SESN2, IRAK2, and IFIT1, whereas representative downregulated genes included KCNMA1, PRKG2, PAEP, and SDHAP1. Hierarchical clustering of representative differentially expressed genes (DEGs) further demonstrated a clear separation between the control and MBP-treated samples, indicating distinct transcriptional profiles following chronic MBP exposure (Figure 2b).

**Figure 3:**
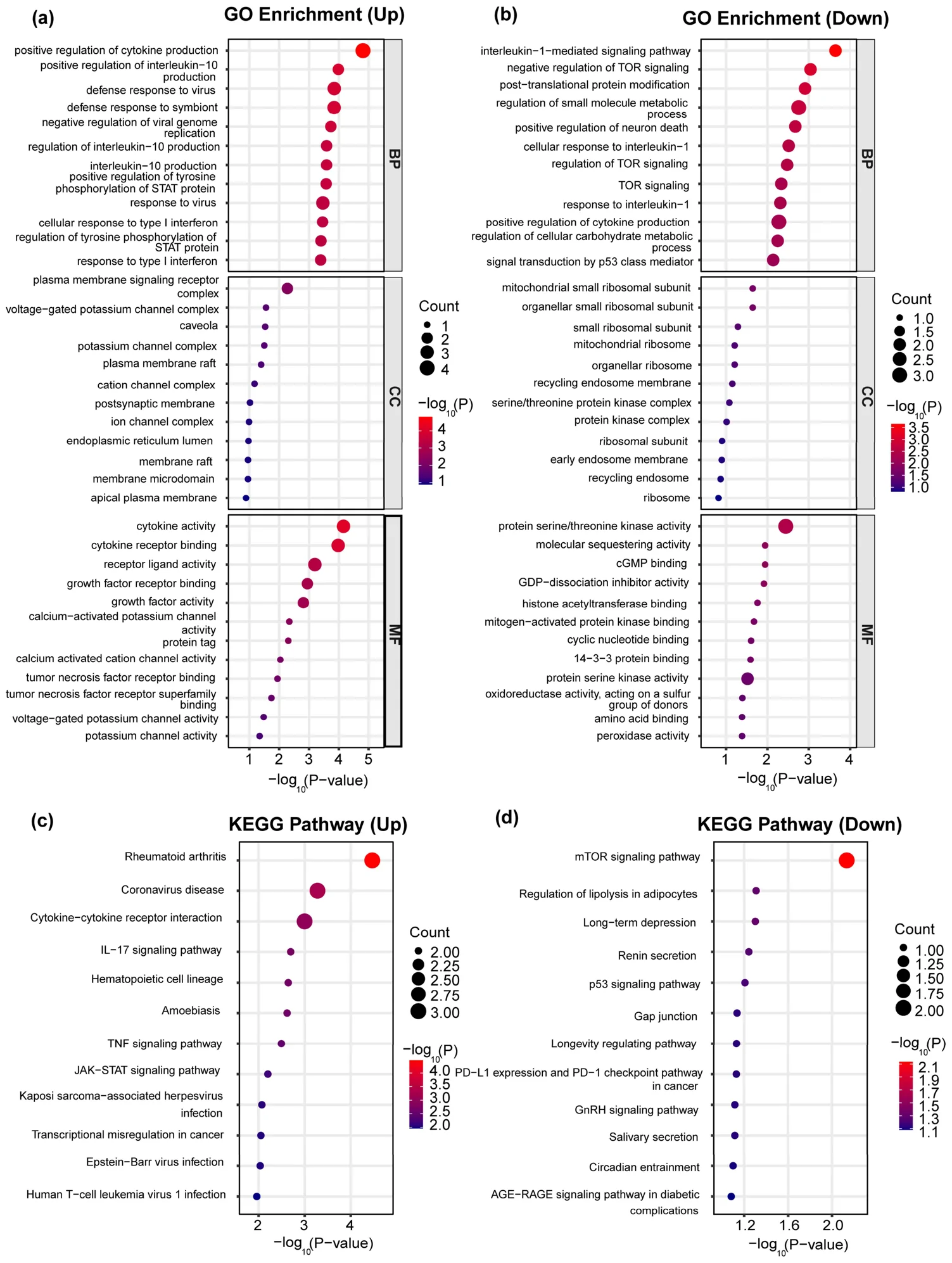
Functional enrichment analysis of differentially expressed genes in MBP-treated cells. (**a**,**b**) GO enrichment analyses of upregulated and downregulated genes, including BP, CC, MP categories. (**c**,**d**) KEGG pathway enrichment analyses of upregulated and downregulated genes, respectively.

To further elucidate the biological significance of these transcriptional alterations, GO and KEGG enrichment analyses were performed. GO enrichment analysis revealed that upregulated DEGs were predominantly associated with interferon-mediated signaling, antiviral response, and cytokine production (Figure 3a), whereas downregulated DEGs were mainly enriched in metabolic processes, kinase activity, and intracellular transport (Figure 3b). KEGG pathway analysis further demonstrated that upregulated DEGs were significantly enriched in multiple inflammation- and cytokine-related signaling pathways, including cytokine–cytokine receptor interaction, the IL-17 signaling pathway, the TNF signaling pathway, and the JAK–STAT signaling pathway (Figure 3c). In contrast, downregulated DEGs were primarily enriched in pathways involved in mTOR signaling, p53 signaling, PD-L1 expression and PD-1 checkpoint pathway in cancer, as well as longevity regulating pathways (Figure 3d).

Collectively, these transcriptomic analyses revealed that chronic MBP exposure induces extensive molecular reprogramming in which inflammatory signaling pathways, rather than canonical stress or DNA damage responses constitute the predominant transcriptional signature. However, while transcriptomic profiling accurately captured the actual gene expression landscape, the upstream regulatory targets and the higher-order network architecture orchestrating this inflammatory response could not be directly inferred from differential expression data alone. Furthermore, transcriptomic analysis alone does not distinguish between directly MBP-perturbed targets and secondary transcriptional adaptations. Therefore, network toxicology analysis was subsequently performed to identify potential upstream targets associated with these transcriptomic alterations.

### 3.3. Integrated Transcriptomic and Network Toxicology Analyses Identify Inflammation-Associated Molecular Signatures Underlying MBP-Induced ATC Progression

**Figure 4:**
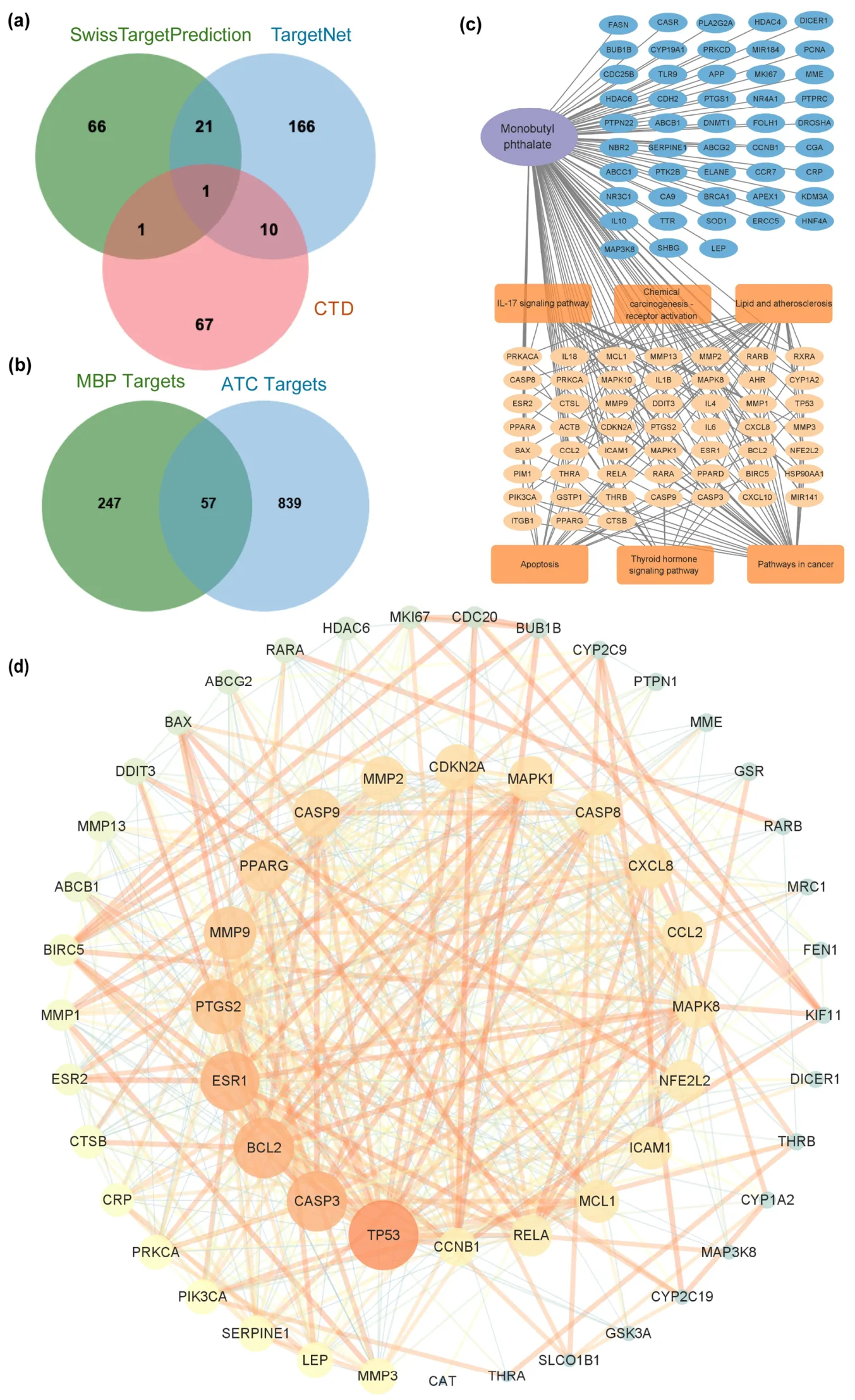
MBP targets overlap with ATC genes. (**a**) Venn diagram showing common targets predicted by SwissTargetPrediction, TargetNet, and CTD. (**b**) Venn diagram showing intersection between MBP targets and ATC genes. (**c**) Network linking overlapping targets to pathways. (**d**) PPI network of the overlapping targets.

To identify the key molecular events underlying the inflammatory transcriptomic response induced by chronic MBP exposure, an integrated strategy combining network toxicology and transcriptomic analyses was employed. Putative MBP-related targets were collected from the TargetNet, Targetnet, CTD, GeneCards, Omim, and SwissTargetPrediction databases (Figure 4a) and intersected with ATC-associated genes, yielding a total of 57 common targets (Figure 4b). Protein–protein interaction (PPI) (Figure S1) analysis of these targets identified several highly connected hub genes, including TP53, CASP3, BCL2, ESR1, PTGS2, PPARG, CASP9, MAPK1, and MMP9, suggesting that MBP may influence multiple biological processes involved in ATC progression (Figure 4c,d).

**Figure 5:**
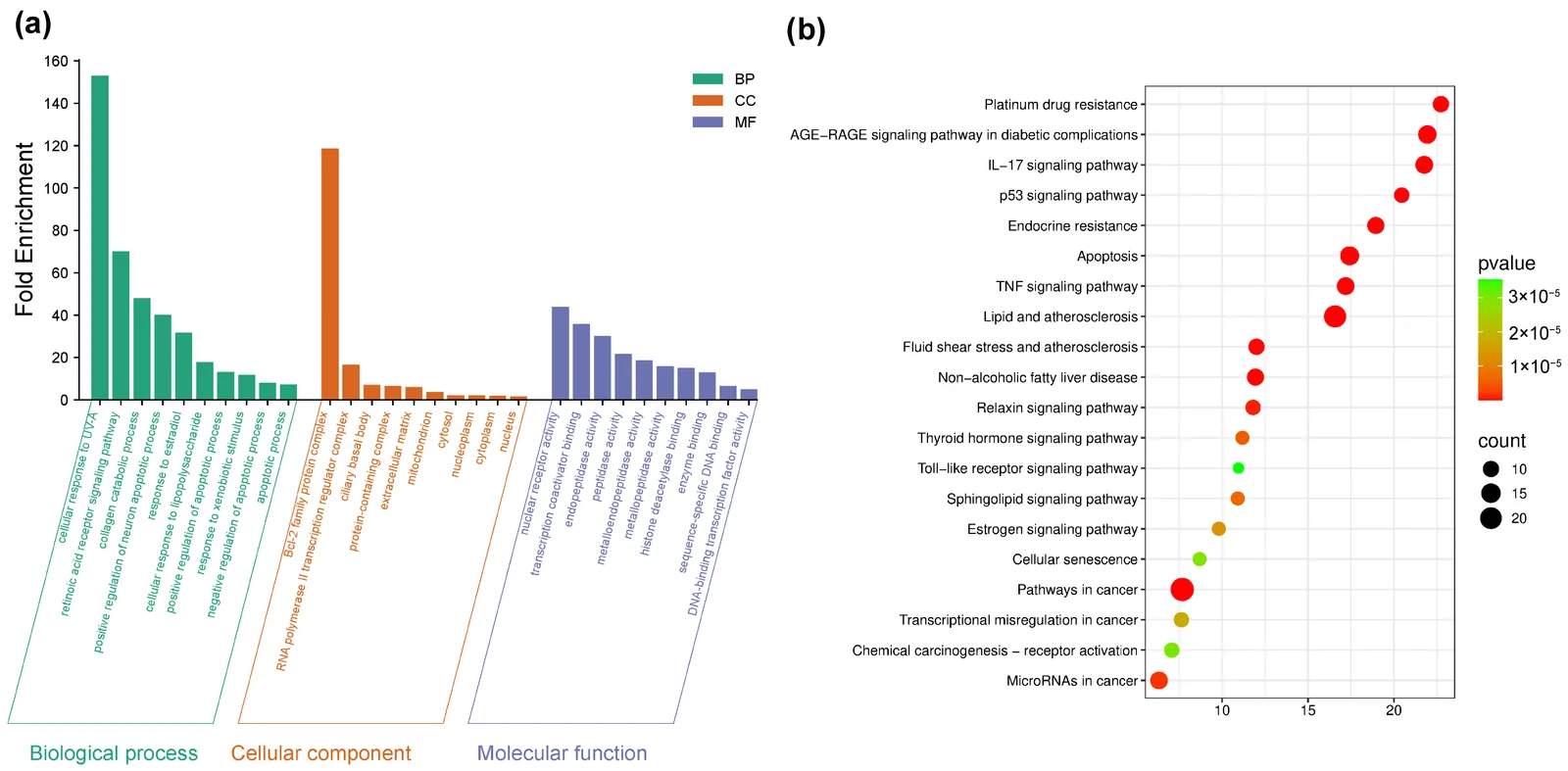
Functional enrichment analysis of potential targets associated with MBP exposure. (**a**) Top 30 GO terms enriched among the candidate target genes. (**b**) Top 20 KEGG pathways enriched among the candidate target genes.

Functional enrichment analysis demonstrated that the common targets were significantly enriched in biological processes associated with apoptotic regulation, xenobiotic response, inflammatory stimulation, and hormone-related signaling. KEGG pathway analysis further revealed significant enrichment of several cancer- and inflammation-related pathways, including the IL-17 signaling pathway, TNF signaling pathway, AGE–RAGE signaling pathway, p53 signaling pathway, apoptosis, thyroid hormone signaling pathway, and pathways in cancer (Figure 5a,b).

Crucially, a notable discrepancy emerged between the network toxicology predictions and the transcriptomic observations. The PPI network and functional enrichment of the predicted common targets prominently featured apoptosis-associated hub genes (TP53, CASP3, BCL2) and apoptosis/p53-related pathways (p53 signaling, apoptosis), suggesting that the computational prediction prioritized apoptosis as a major potential response to MBP exposure. However, this stood in marked contrast to the transcriptomic data, which showed predominant activation of inflammatory pathways and, notably, suppression rather than activation of p53/mTOR signaling in chronically exposed cells. This apparent decoupling between in silico prediction—which largely reflects acute, high-dose perturbation data compiled in existing databases—and the actual transcriptional response under chronic, low-dose exposure conditions indicated that chronic MBP exposure does not simply activate the predicted apoptotic machinery, but rather engages a distinct, inflammation-dominant adaptive program. This discrepancy prompted us to integrate the two datasets to identify the convergence—rather than divergence—between predicted targets and observed transcriptomic changes.

**Figure 6:**
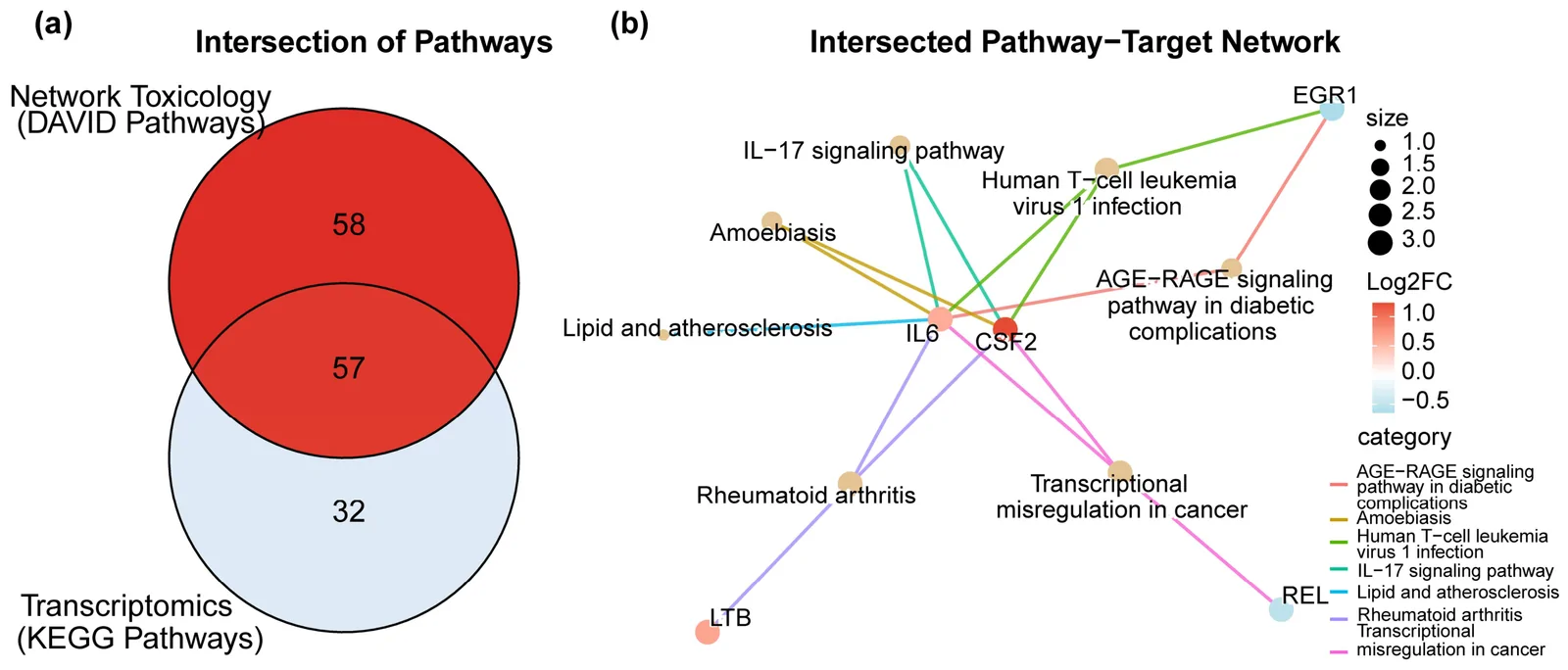
Integrated analysis of the molecular mechanisms associated with MBP-induced cellular responses. (**a**) Venn diagram illustrating the overlapping pathways identified by transcriptomic and network toxicology analyses. (**b**) Pathway–target interaction network of the shared pathways and representative target genes identified through integrated analysis.

To prioritize the most biologically relevant pathways associated with chronic MBP exposure, KEGG enrichment results derived from network toxicology were integrated with transcriptomic profiling. Among the 130 enriched pathways predicted by network toxicology and the 89 pathways identified by RNA sequencing, 57 common pathways were identified (Figure 6a). Construction of a pathway–target interaction network further revealed that IL6 and CSF2 exhibited the highest connectivity, linking multiple inflammation-associated pathways, including the IL-17 signaling pathway, AGE–RAGE signaling pathway in diabetic complications, lipid and atherosclerosis, rheumatoid arthritis, and transcriptional misregulation in cancer (Figure 6b). Rather than converging on a single signaling cascade, these hub genes interconnected multiple inflammatory pathways, suggesting that chronic MBP exposure preferentially remodels an inflammation-associated molecular network.

Taken together, the integrated analyses consistently converged on inflammation-associated signaling as the predominant molecular signature of chronic MBP exposure. Notably, network topology shifted from apoptosis-centered hub genes (TP53, CASP3, BCL2) in the PPI network—representing the computational prediction—to inflammation-centered hub genes (IL6, CSF2) in the integrated pathway–target network—representing the convergence of prediction and biological reality. Among the identified hub genes, IL6 and CSF2 emerged as central regulatory nodes linking multiple inflammatory pathways, thereby providing candidate molecular mediators for subsequent experimental validation.

### 3.4. Experimental Validation Supports Inflammation-Associated Dysregulation of Apoptosis-Related Signaling Following Chronic MBP Exposure

To experimentally evaluate the integrated bioinformatic predictions, five representative targets were selected to cover three core biological processes implicated by our integrated analyses: inflammatory signaling (IL6), apoptosis regulation (TP53, CASP3, BCL2), and metabolic/inflammatory crosstalk (PPARG). PPARG was of particular interest given its established role in integrating metabolic and inflammatory signaling, which may be relevant to the metabolic pathway alterations observed in the transcriptomic data.

**Figure 7:**
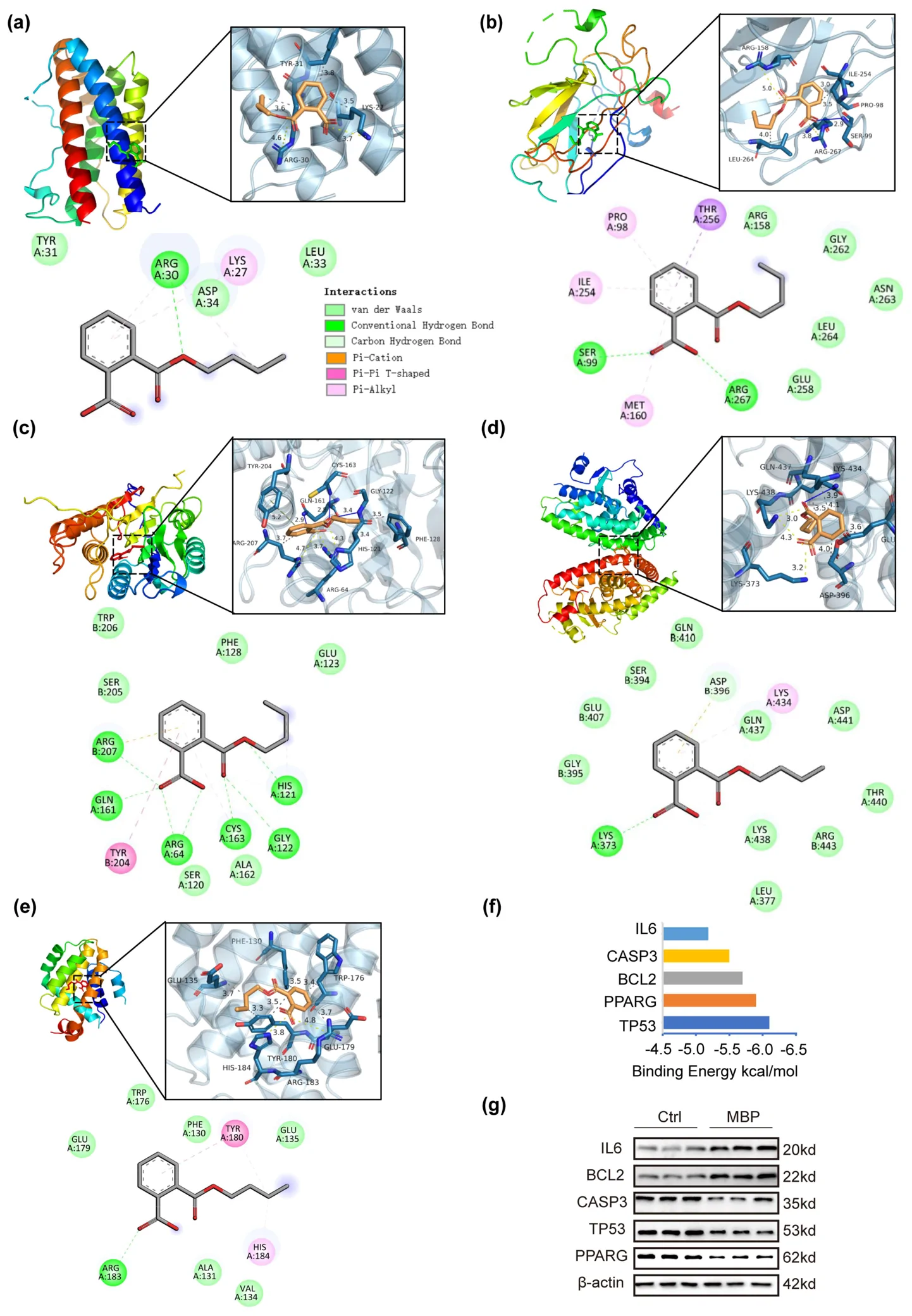
Molecular docking and Western blot validation of MBP interactions with key hub proteins in ATC progression. (**a**–**e**) Docking conformations of MBP with IL6, TP53, CASP3, PPARG, and BCL2. (**f**) Binding affinity comparison between MBP and the five targets’ proteins. (**g**) Western blot showing protein expression changes in the five hub proteins upon MBP exposure.

To further evaluate the potential interactions between MBP and the identified candidate proteins, molecular docking analysis was performed using TP53, CASP3, BCL2, IL6, and PPARG. MBP exhibited favorable binding affinities toward all five proteins, with binding energies ranging from −5.2 to −6.1 kcal/mol (Figure 7a–e). Among them, TP53 showed the strongest predicted binding affinity (−6.1 kcal/mol), followed by PPARG (−5.9 kcal/mol), BCL2 (−5.7 kcal/mol), CASP3 (−5.5 kcal/mol), and IL6 (−5.2 kcal/mol), indicating that MBP possesses the potential to interact with multiple proteins involved in inflammation, apoptosis, and metabolic regulation. In molecular docking studies, binding energies lower than −5.0 kcal/mol are generally considered indicative of meaningful ligand–protein interactions, suggesting that MBP exhibits promising binding potential with these targets. The docking analysis was performed using MBP as the ligand molecule interacting with the selected target proteins following a conventional molecular docking workflow, and no competitive docking assays with endogenous ligands were performed. Therefore, the docking results were interpreted as computational predictions of possible interaction modes rather than direct evidence of physical binding.

To experimentally validate the integrated bioinformatic predictions, the protein expression levels of representative hub molecules were examined by Western blotting (Figure 7g). Densitometric analysis was performed based on three independent biological replicates with statistical evaluation. (Figure S2). Consistent with the integrated transcriptomic and network analyses, chronic MBP exposure significantly increased IL6 protein expression compared with the control group (*p* < 0.05). In contrast, the expression of TP53 and the executioner caspase CASP3 was markedly decreased following chronic MBP exposure (*p* < 0.05), whereas the anti-apoptotic protein BCL2 was significantly upregulated (*p* < 0.05). In addition, PPARG expression was significantly reduced in MBP-treated cells (*p* < 0.05), suggesting disruption of pathways associated with metabolic homeostasis and inflammatory regulation.

Overall, the experimental findings were highly consistent with the integrated bioinformatic analyses. Chronic MBP exposure was associated with enhanced inflammatory signaling, represented by increased IL6 expression, together with suppression of apoptosis-related signaling, as reflected by decreased TP53 and CASP3 expression and elevated BCL2 levels. These findings support the concept that chronic environmentally relevant MBP exposure promotes ATC progression through coordinated remodeling of inflammation- and apoptosis-associated molecular networks, with IL6 functioning as a key inflammatory hub and the TP53–CASP3–BCL2 axis representing a critical node of apoptosis dysregulation.

## 4. Discussion

Our study demonstrates that chronic exposure to environmentally relevant concentrations of MBP promotes the malignant phenotype of CAL-62 and is accompanied by coordinated alterations in inflammatory and apoptosis-related signaling. By integrating network toxicology, transcriptomic profiling, molecular docking, and protein validation, we identified an inflammation-dominant molecular signature characterized by increased IL6 expression together with reduced TP53 and CASP3 expression and elevated BCL2 levels. Rather than affecting a single signaling cascade, our findings suggest that chronic MBP exposure is associated with coordinated remodeling of interconnected inflammatory and apoptosis-related molecular networks that collectively favor ATC progression.

Most mechanistic studies investigating phthalate toxicity have relied on acute or relatively high-dose exposure models [27,28]. Although these studies have identified alterations in oxidative stress, endocrine signaling, or individual inflammatory pathways, they may not fully reflect the biological consequences of chronic low-dose environmental exposure. Moreover, cancer cells may be particularly susceptible to chronic low-dose environmental toxicants, as previously reported in MBP-exposed hepatocellular carcinoma models [29]. The exposure concentration (10 nM) was selected to represent an environmentally relevant low-dose exposure based on previous human biomonitoring studies reporting the presence of MBP and related phthalate metabolites in human biological samples, including serum, breast milk, and urine [30,31,32]. This concentration was therefore chosen to better mimic chronic environmental exposure rather than conventional acute high-dose toxicological conditions. In this study, continuous exposure to 10 nM MBP for approximately 3 months significantly enhanced proliferation, colony formation, and tumorsphere formation of CAL-62 cells. These observations suggest that prolonged exposure to environmentally relevant MBP concentrations may influence tumor cell behavior even in the absence of overt cytotoxicity, emphasizing the necessity of incorporating environmentally relevant chronic exposure paradigms into mechanistic toxicological studies.

Transcriptomic analysis revealed that inflammatory signaling represented the predominant transcriptional response following chronic MBP exposure. This conclusion was supported by the observation that the majority of significantly enriched upregulated KEGG pathways were associated with cytokine-mediated inflammatory signaling, including cytokine–cytokine receptor interaction, IL-17, TNF, and JAK–STAT signaling pathways, whereas pathways related to p53 signaling and mTOR signaling were comparatively downregulated. Chronic inflammation is widely recognized as an important component of tumor progression because inflammatory cytokines can promote proliferation, survival, angiogenesis, and therapeutic resistance [33,34]. The enrichment of multiple cytokine-associated pathways observed in this study therefore suggests that persistent inflammatory signaling may contribute to the enhanced malignant phenotype induced by chronic MBP exposure [29].

One of the most notable findings of this study is the difference between the molecular features highlighted by network toxicology and those identified by transcriptomic profiling. Network toxicology prioritized apoptosis-associated hub genes such as TP53, CASP3, and BCL2 based on protein interaction topology, whereas the integrated pathway analysis identified IL6 and CSF2 as the most highly connected nodes linking multiple enriched inflammatory pathways. Rather than representing contradictory results, these findings likely reflect the complementary information provided by the two analytical approaches. Network toxicology predicts potential molecular targets based on existing biological knowledge, whereas transcriptomic profiling captures the gene expression landscape under the specific experimental conditions. Integrating these approaches therefore enabled the identification of molecular pathways that were both computationally predicted and experimentally supported, thereby highlighting inflammation-associated signaling as the dominant biological feature associated with chronic MBP exposure. Therefore, chronic environmental toxicology studies should not rely solely on database-driven predictions. Instead, the integration of computational approaches with omics-based profiling and experimental validation provides a more reliable framework for identifying biologically relevant molecular alterations and elucidating mechanisms associated with long-term low-dose exposure. This discrepancy also highlights an important consideration regarding the interpretation of database-derived toxicological predictions. Most currently available toxicology databases are constructed based on accumulated experimental evidence from conventional toxicity studies, which frequently involve acute exposure conditions, relatively high concentrations, or targeted investigations of individual molecular events. Therefore, database-based predictions may preferentially reflect historically recognized toxicity-associated targets and may not fully capture adaptive cellular responses or molecular network remodeling induced by prolonged exposure to environmentally relevant concentrations. In the context of chronic low-dose exposure, biological responses may involve dynamic reprogramming of interconnected pathways rather than direct activation or inhibition of predefined targets. Thus, computational predictions generated from existing databases should be considered as hypothesis-generating tools rather than definitive representations of chronic exposure responses.

Among the identified regulatory molecules, consistent with its role as a central hub in the integrated pathway–target network, The central network position of IL6 together with its increased protein expression further supports the conclusion that inflammatory signaling constitutes the dominant molecular response following chronic MBP exposure [35,36]. Serum IL-6 levels are significantly elevated in thyroid cancer patients and are associated with poor overall survival and disease aggressiveness [37]. IL-6 has been shown to promote thyroid cancer cell proliferation, dedifferentiation, and inhibition of apoptosis through activation of the IL-6/JAK2/STAT3 signaling pathway [38]. Moreover, the IL6/STAT3 axis mediates resistance to BRAF inhibitors in thyroid carcinoma cells, and targeting IL6 signaling has been proposed as a strategy to improve therapeutic sensitivity [39]. In anaplastic thyroid carcinoma specifically, high levels of circulating and monocyte-derived IL-6 have been reported, and ATC cell lines have been shown to secrete IL-6 [40,41]. The observation that IL6 was connected to multiple enriched pathways rather than a single signaling cascade suggests that inflammatory signaling may function as a coordinated regulatory network under chronic MBP exposure. This network-level perspective is further supported by the enrichment of IL-17, TNF, and cytokine-related pathways observed in the transcriptomic analysis. In contrast to the activation of inflammatory signaling, apoptosis-related molecules exhibited an opposite pattern. Protein expression analysis demonstrated decreased TP53 and CASP3 expression together with increased BCL2 expression following chronic MBP exposure. TP53 is a key tumor suppressor that maintains genomic integrity by inducing cell-cycle arrest and apoptosis in response to cellular stress. Reduced TP53 expression may impair these protective mechanisms, thereby allowing damaged cells to evade apoptosis and acquire survival and proliferative advantages. In ATC, TP53 alterations are frequently associated with aggressive tumor behavior, including rapid proliferation, dedifferentiation, genomic instability, and therapeutic resistance. These findings are consistent with suppression of apoptosis-associated signaling and may contribute to the increased proliferative and clonogenic capacities observed in chronically exposed cells. This pattern is also consistent with the transcriptomic observation that p53 signaling was enriched among downregulated pathways, further corroborating the convergence of transcriptional and protein-level findings. Although the present study does not establish a direct causal role for TP53 suppression in mediating MBP-induced ATC progression, the coordinated decrease in TP53 and CASP3 together with increased BCL2 expression suggests attenuation of TP53-associated apoptotic regulation, which may represent an important molecular event contributing to the malignant phenotype induced by chronic MBP exposure.

PPARG was also significantly downregulated following chronic MBP exposure. In addition to its established role in lipid metabolism, PPARG has been implicated in regulating inflammatory responses and cellular differentiation. Reduced PPARG expression has been associated with enhanced inflammatory activity in multiple disease models [42]. Although the functional significance of PPARG downregulation was not directly investigated in our study, its consistent decrease at the protein level suggests that it may participate in the broader molecular alterations associated with chronic MBP exposure and warrants further investigation.

This study has several strengths. First, the chronic exposure model using environmentally relevant MBP concentrations more closely reflects realistic human exposure scenarios than conventional acute exposure studies. Second, the integration of network toxicology with transcriptomic profiling enabled systematic prioritization of biologically relevant pathways by leveraging the complementarity between computational prediction and expression profiling. Third, molecular docking and protein validation provided independent experimental support for the integrated toxicogenomic findings, strengthening the robustness of the proposed molecular framework. Our findings further emphasize the necessity of integrating computational prediction with experimental validation, particularly in chronic exposure studies. While network toxicology provides valuable insights into potential molecular targets and biological networks, transcriptomic profiling and experimental verification are essential to determine whether these predicted alterations are actively engaged under specific exposure scenarios. Therefore, future chronic environmental toxicology studies should adopt integrative strategies combining database-based prediction, omics-based profiling, and functional validation to improve the accuracy and biological relevance of mechanistic interpretation. Several limitations should also be acknowledged. Although molecular docking analysis suggested potential interactions between MBP and key target proteins, these computational predictions do not directly demonstrate physical binding. Further experimental validation using biophysical approaches, such as SPR or ITC, is required to confirm the binding affinity and interaction mechanisms. Functional studies targeting IL6, PPARG, or apoptosis-related molecules were not performed; therefore, causal relationships among these signaling components remain to be established. In addition, although CAL-62 is a well-established ATC cell model widely used for mechanistic investigations, a single cell line cannot fully capture the molecular heterogeneity of ATC. Furthermore, while PTC represents the most prevalent thyroid cancer subtype, the present study focused on ATC because of its highly aggressive biological characteristics and unmet therapeutic challenges. We also acknowledge that we did not perform dedicated assays to systematically evaluate cellular senescence or long-term phenotypic drift during the extended culture period. However, throughout the chronic exposure process, control and MBP-treated cells were maintained under identical culture conditions and were routinely monitored for morphology and growth characteristics, with no obvious changes observed in the control group. Future studies incorporating senescence-associated markers, such as SA-β-gal staining and p16/p21 expression analysis, will be necessary to further validate the stability of long-term exposure models. Future studies incorporating additional ATC models, other thyroid cancer subtypes including PTC, and in vivo validation will be necessary to determine the broader relevance of MBP-associated molecular alterations.

## 5. Conclusions

The present study demonstrated that chronic exposure to environmentally relevant concentrations of MBP promotes the malignant progression of CAL-62. By integrating network toxicology with transcriptomic profiling, molecular docking, and protein validation, we identified a coordinated molecular alteration characterized by inflammation-associated network remodeling accompanied by suppression of apoptosis-related signaling. Specifically, integrated analyses consistently prioritized IL6 as a central inflammatory hub, whereas TP53, CASP3, BCL2, and PPARG were experimentally validated as representative molecules associated with dysregulated apoptosis- and metabolism-related signaling. More importantly, this study highlights the value of integrating computational prediction with experimental multi-omics data to distinguish biologically relevant molecular networks from predicted candidate targets under chronic environmental exposure conditions. Rather than acting through a single signaling pathway, chronic MBP exposure appears to be associated with coordinated alterations across interconnected inflammatory and apoptosis-related signaling networks that collectively favor malignant progression.

These findings provided mechanistic insights into the potential role of long-term exposure to environmental phthalate metabolites in ATC progression and underscore the importance of incorporating environmentally relevant chronic exposure models into toxicological investigations of endocrine-disrupting chemicals. Overall, this study provides a systems-level framework for understanding how chronic exposure to environmental contaminants reshapes tumor-associated molecular networks and offers a foundation for future mechanistic investigations into environmentally associated cancer progression.

## Data Availability

The original contributions presented in this study are included in the article/Supplementary Material. Further inquiries can be directed to the corresponding authors.

## Supplementary Materials

The following supporting information can be downloaded at: https://www.mdpi.com/article/10.3390/biomedicines14081755/s1, Figure S1. The hub gene subnetwork. The subnetwork shows the interactions among the identified hub genes and their closely connected neighbors. Figure S2. Quantitative analysis of Western blot results for representative hub proteins following chronic MBP exposure.

## Abbreviations

The following abbreviations are used in this manuscript:

MBP	Monobutyl phthalate
ATC	Anaplastic thyroid carcinoma
EDCs	Endocrine disrupting chemicals
CCK-8	Counting Kit-8
CTD	Comparative Toxicogenomics Database
GO	Gene Ontology
KEGG	Kyoto Encyclopedia of Genes and Genomes
BP	biological process
MF	molecular function
CC	cellular component
