# Supplementary material for: Chronic Monobutyl Phthalate Exposure Promotes Anaplastic Thyroid Cancer Progression Through Inflammatory Signaling Dysregulation: Integrated Transcriptomic and Network Toxicology Analyses
Source: Biomedicines. 2026 Aug 4;14(8):1755. doi: 10.3390/biomedicines14081755 (PMC13509629; doi:10.3390/biomedicines14081755)
Supplement: Supplementary file 1 [file biomedicines-14-01755-s001.zip › biomedicines-4465097-supplementary.pdf]

Supplementary Materials

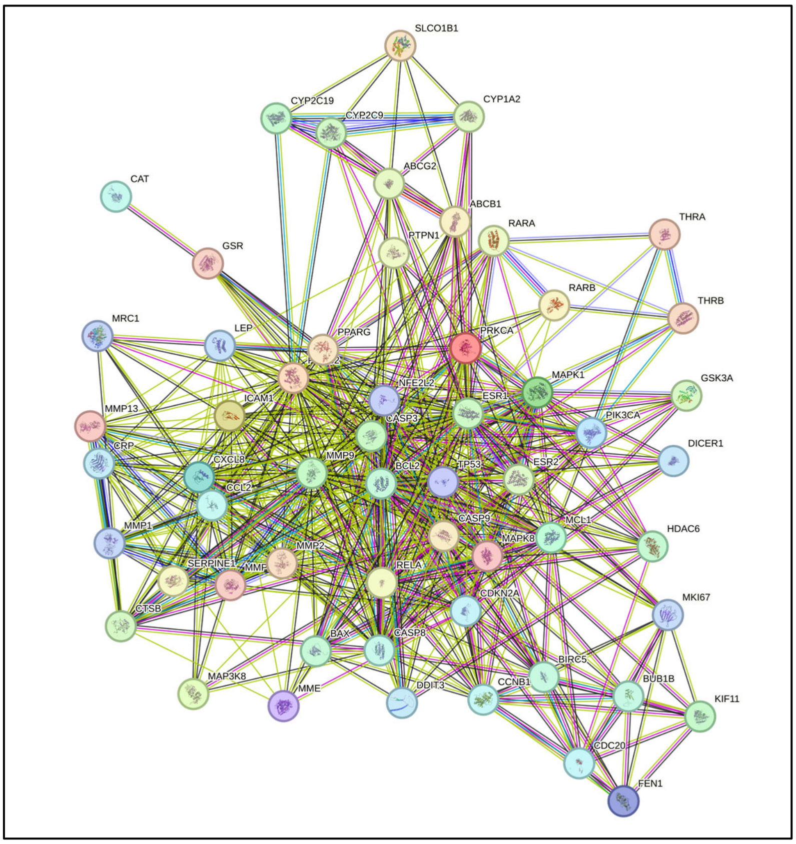

Figure S1. The hub gene subnetwork. The subnetwork shows the interactions among the identified hub genes and their closely connected neighbors.

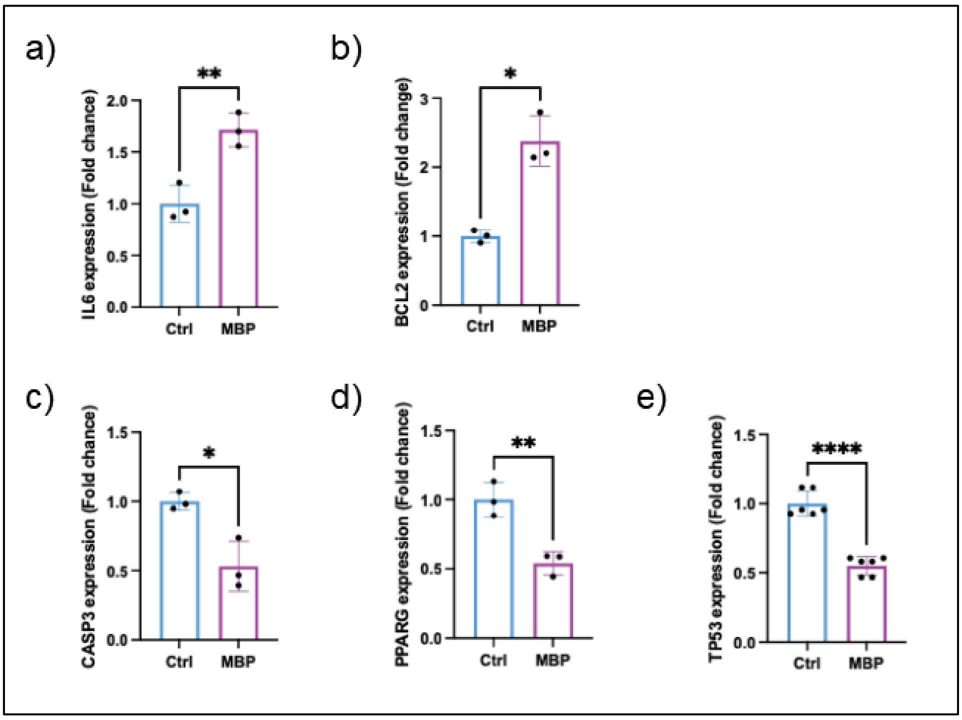

Figure S2. Quantitative analysis of Western blot results for representative hub proteins following chronic MBP exposure.

*Relative protein expression levels of IL6, TP53, CASP3, BCL2, and PPARG were quantified by densitometric analysis and normalized to β-actin. Data were obtained from three independent biological replicates and are presented as mean ± SD. $P < 0.05$ versus the control group.
